# Notes on Ophthalmology

**Published:** 1875-11

**Authors:** Lucien Howe


### MEDICAL NOTES.

ART. 1—*Notes on Ophthalmology.*—By LUCIEN HOWE, M. D.

1. EXAMINATION OF COLORED SCHOOL CHILDEN'S EYES, BY PETER A. CALLAN, M. D.—Another contribution is here made to a subject which is receiving well deserved attention in this country. It will be remembered that the extensive investigations of Cohn in Germany, and Erismann in Russia, have proved that the habits of school life tend strongly to produce nearsightedness. Within two years past, similar examinations have been made in New York, Brooklyn, and Cincinnati, the results of which are embodied in a paper read at the last meeting of the American Social Science Association, in Detroit, by Dr. Webster, of New York. It was found, for instance, that while the district schools contained less than 14 per cent. of near sighted pupils, that proportion increased steadily through the intermediate, high and normal schools, to over 22 per cent. in the latter. The immediate cause of this is undoubtedly the neglect of hygienic rules relating to the position of the body while studying, to the amount of light supplied, and several other apparently trivial matters.

But a predisposing and not less important cause is the hereditary tendency to nearsightedness among children of the present day, derived from generations of already educated parents. It is this phase of the subject which Dr. Callan presents in an interesting light. His examinations were made upon a set of children subjected in an equal degree as the whites, to all the influences which are known to develop nearsightedness, with the exception of belonging to a race which has not until now enjoyed any educational advantages. The result is significant and instructive. While other schools for white children in this country have shown a proportion, as stated, of from 13,3 per cent. to 22,8 per cent. of nearsighted pupils, among the negroes of the same grades and ages, there was found to be an average of only 2,6 per cent. Although such a comparison is in many respects unfair, it holds good in the main, and gives some idea of how great is the influence of the hereditary tendency in the development of nearsightedness.

If this disease never amounted to anything more than the inconvenience usually experienced, the whole matter might be rather one of interest than importance. But unfortunately it sometimes assumes that dangerous form called "progressive" nearsightedness, or myopa, a disease the diagnosis of which is easy enough, but the successful treatment is a matter of extreme difficulty.

Some very satisfactory results were, however, reported at the meeting of the Opthalmological Society, in 1874, in a paper.

II. ON THE ATROPINE TREATMENT OF ACQUIRED AND PROGRESSIVE MYOPIA, WITH A TABLE OF CASES. BY HASKET DERBY, M. D.—Recognizing the fact that in extreme degrees of Myopia there is generally "a species of spasm of the accommodation," Dr. Derby sought to overcome this by the use of Atropine. Solutions of the sulphate of various strength were used, but in all cases it was sufficiently concentrated to produce complete dilatation of the pupil. In order to overcome the spasm entirely, the treatment was continued from two, to eight or ten weeks, as the case required.

As a result he found that in 5,52 per cent. there was definite improvement; in 8,9 per cent. the myopia was arrested; in the remainder the myopia either progressed or the result was unknown.

III. INVESTIGATIONS CONCERNING THE PHYSIOLOGICAL ACTION OF ATROPIA AND PHYSOSTIGMIN ON THE HEART AND PUPIL.—ROSSBACH. *Verhandl. der physical-medicin, Ges. in Wirzburg.* Bd. V. Heft. 1, pag 1. bis. 79.)

In regard to the comparative action of Atropia and Calabar bean, it has been commonly supposed that they are antagonistic, and so they seem to be practically. But exact experiment shows that the effect of both is first to contract and then dilate the pupil. With Atropia it is only the last stage, and with Calabar bean the first, which is principally apparent.

IV. HOW CALOMEL ACTS WHEN DUSTED IN THE EYE. KAMOMERER.—(*Virchow's Archiv. Bd* 59, *page* 467.)

Light has recently been thrown on the action of another substance much used in diseases of the eye. That is Calomel. Phlyctenular forms of Conjunctivitis and Corneitis often yield readily to this when applied locally, as a result, it was supposed, of the mechanical irritation produced. But an examination of the urine of patients under such treatment shows that the mild chloride is changed into the bichloride, and acts as such.

V. CONCERNING IRIDOTOMIE. KRUGER.—(*Sitzungscericht der Opthalmologischen Gesellschaft*, 1874.)—ON IRIDOTOMY—GROSSMANN.—(*Klmische Monatsblatter fur Augenheilkunde*, April, 1875.) IRIDOTOMY AND ITS APPLICABILITY TO CERTAIN DEFECTS OF THE EYE—A. W. CALHOUN, M. D., 1875.

The literature relating to this operation increases, as its satisfactory results become more numerous. The idea is, to make a simple slit across the iris, and allow the retracting edges of the wound to form the pupil. To this end the same corneal incision is made as in Iridectomy, and through it there is introduced a kind of forcep-like scissors to make the desired cut. Such a procedure can be substituted for iridectomy whenever the latter would be indicated in cases where an opaque cornea hides the real pupil, or where that is filled by an exudation, as in iritis following cataract extraction. It has the advantage over iridectomy of offering less violence to the iris, that structure being cut *into* instead of being cut *out*. But another reason for preference given to it is, that there is no such flurred indistinctness of vision produced, as often follows iridectomy. The rays of light passing through a smaller opening are brought to a more sharply defined focus on the retina.

